# Association Between Lack of Access to a Neighborhood Park and High Blood Pressure in the Philadelphia Metropolitan Area

**DOI:** 10.5888/pcd20.230098

**Published:** 2023-11-02

**Authors:** Stephanie Kjelstrom, Richard W. Hass, Russell K. McIntire

**Affiliations:** 1 Thomas Jefferson University, College of Population Health, Philadelphia, Pennsylvania; 2 Main Line Health Center for Population Health Research, Wynnewood, Pennsylvania; 3 Lehigh University, College of Health, Bethlehem, Pennsylvania

## Abstract

**Introduction:**

Studies have shown a lower risk of high blood pressure (HBP) among people who live near parks; however, little information exists on how feeling safe and comfortable visiting the park affects blood pressure. We identified associations between neighborhood park access, comfort visiting a park, and HBP to understand how these factors may contribute to disparities in HBP prevalence.

**Methods:**

The 2018 Southeastern Pennsylvania Household Health Survey of 3,600 residents in the Philadelphia metropolitan area asked if respondents had ever been told they had HBP and whether they had a neighborhood park or outdoor space that they were comfortable visiting during the day. To assess the association between park access and HBP, we built multilevel logistic models to account for variation in HBP by zip code. We examined the effect modification of perceptions of park access (having a neighborhood park, not having a neighborhood park, or having a neighborhood park but not comfortable visiting it) and HBP by race, education, and poverty status.

**Results:**

Both not having a neighborhood park and having a park but not feeling comfortable visiting it were associated with higher unadjusted odds of HBP, 70% and 90%, respectively, compared with having a neighborhood park. Adjusted odds ratios for the lack-of-park responses remained significant (no neighborhood park, adjusted odds ratio [aOR] = 1.4; 95% CI, 1.1–1.7; neighborhood park but not comfortable visiting, aOR = 1.4; 95% CI, 1.03–2.0). A significant gradient was observed for Black respondents compared with White respondents with odds of HBP increasing by perceptions of park access (aOR = 1.95 for people with a park; aOR = 2.69 for those with no park; aOR = 3.5 for people with a park that they are not comfortable visiting).

**Conclusion:**

Even accounting for other risk factors for HBP, not having a neighborhood park or not feeling comfortable visiting one may influence individual HBP. Neighborhood factors that deter park access may contribute to racial disparities in HBP.

**Summary**

**What is already known on this topic?**

Access to neighborhood green spaces such as parks may reduce high blood pressure.

**What is added by this report?**

Although lowered blood pressure is related to access to parks, not feeling comfortable visiting the park may negate its good effects.

**What are the implications for public health practice?**

Both having access to a nearby park and feeling safe and comfortable visiting that park may be important predictors of high blood pressure.

## Introduction

Parks play a role in promoting physical activity and social connectedness within communities. Physical activity is essential for preventing and controlling risk factors and chronic diseases, such as diabetes, high blood pressure (HBP), cardiovascular disease (CVD), obesity, cancer, and depression (1). Likewise, social connectedness improves mental health, promotes healthy behaviors, and increases life expectancy (2,3). Studies have examined the presence of green spaces, including open-space parks, tree cover, and vegetation and their association with health outcomes. Some studies indicate that proximity to green spaces is linked to a lower risk of HBP, improved diabetes outcomes, and reduced obesity (4–8). However, people’s use of parks depends on feeling safe and comfortable in their neighborhood. Studies have shown that factors such as the presence of walking paths and lighting, along with crime rates, can affect the use of parks for physical activity (9–13). No studies to date have evaluated the association between feeling comfortable visiting a park and the risk of chronic diseases such as HBP.

In the United States, race and socioeconomic status (SES) are risk factors for cardiovascular disease, and Black residents and those of lower SES have higher rates of HBP (14–16). Additionally, health disparities are evident in certain areas, such as historically redlined neighborhoods. Philadelphia County, Pennsylvania, has a lower health ranking than other counties of similar size, and its historically redlined neighborhoods continue to be racially segregated, have lower financial investment, and have worse health outcomes (17,18). For example, areas of primarily minority residents in Philadelphia have higher rates of diabetes, infant mortality, HBP, CVD deaths, and all-cause mortality compared with predominantly White areas and counties along Philadelphia County’s border (19).

The concept of “equigenesis” (when something in the environment disrupts the usual relationship between economic disadvantage and a poor health outcome, making lower and higher economic status groups more equal) proposes that access to green spaces can reduce neighborhood health disparities (20). A recent study measured tree cover and vegetation by using both objective satellite imagery and perceived access through survey data from the Southeastern Pennsylvania Household Health Survey (SEPHHS). Perceived access was measured by using a question that asked participants whether there was a park in their neighborhood. Findings indicated that perceived access to green space was protective of HBP and had an equigenic effect in low-income neighborhoods (21). However, Koh et al (22) used the same data and did not observe such an effect on HBP disparities. Overall, green spaces such as neighborhood parks can reduce HBP, but questions remain about whether feeling comfortable and safe visiting a park is associated with a lower risk of HBP and whether parks can mitigate HBP disparities.

Our study focused on parks as green spaces and further examined the association between perceived park access, feeling comfortable visiting a park, and HBP. We also used individual-level data to explore any equigenic effects. We were interested in whether an equigenic effect was present among Black, low-income, or less educated participants in SEPHHS.

## Methods

We used data from the 2018 SEPHHS, a cross-sectional, biennial health survey conducted via telephone (random-digit dialing), by using zip-code–stratified clusters, across southeastern Pennsylvania counties (Bucks, Chester, Delaware, Montgomery, and Philadelphia) (23). Response rates overall were 7.8% for cellular telephones and 6.3% for landlines and varied by source from 2% for random-digit dialing to 38% for re-contacts from past surveys (23). In 2018, the survey included a question about park access: “Is there a park or other outdoor space in your neighborhood that you’re comfortable visiting during the day?” The 3 answer options were 1) “Yes, there is an outdoor space or park that you are comfortable visiting,” 2) “No, there is no park in your neighborhood,” or 3) “No, there is a park in your neighborhood, but you are not comfortable visiting it.” The parks-access question was administered via Form A of the SEPHHS to half of the sampled survey participants, a total of 3,605 participants. HBP was determined by answers to the question, “Has a doctor ever told you that you have high blood pressure?” For the purposes of the current analysis, answers of “no” and “no, but borderline high or pre-hypertensive” were considered to indicate an absence of HBP, and “yes” indicated someone with HBP. To determine whether equigenic relationships were present, we evaluated the effect modification of HBP and park access by race, educational level, and poverty status (annual income above or below 150% of the federal poverty level). Institutional review board approval was not sought for this study because the data are archival and deidentified.

### Statistical analysis

We used standard descriptive statistics to summarize continuous and categorical variables. We compared the demographics, comorbidities, and other characteristics of participants with HBP to those without HBP by using χ^2^ tests of independence for categorical variables and 2-sample *t-*tests for continuous variables. We included variables that are identified in the literature as risk factors for HBP: age, sex, race (categorized as Black, White, or other), education level (less than high school graduate, high school graduate, some college, college graduate, or graduate school), poverty status, marital status (married, single, divorced, or widowed), and social capital (low, medium, high) (13,21,22). Social capital was measured with 5 questions about the characteristics of a participant’s neighborhood; lower scores indicated less community togetherness and belonging. We categorized race into 3 categories because of the small number of participants who identified as neither White nor Black. We also used race as a social construct for racism, as a possible explanation for racial disparities in HBP prevalence. In addition, we added the following comorbidities: diabetes, asthma, obesity, and any mental health disorder. These were marked as present if a participant indicated they had ever had the condition and included in analyses because they are associated with higher rates of HBP (24,25). Finally, to account for the effect of behaviors on HBP (25), we added 3 variables about smoking, diet, and exercise; whether a person had ever smoked; whether someone ate fruits and vegetables 0 to 2 times a day or 3 or more times a day; and whether they exercised at least 3 times a week.

We built logistic multilevel models with 2 levels, individual-level variables and zip code, to estimate the association between the perception of park access and HBP, with HBP as the dependent variable. Our analysis included several models: 1) the random-intercept or “empty” model with only zip code to assess the need for random effects (26), 2) a model that included only the park access variable, 3) a model that included all possible confounders (Table 1), and 4) the final model, which was built by using a backward selection method with Akaike information criterion and *P* < .05 as a criterion for inclusion. The covariables included in the final model were age, sex, race, poverty status, education, ever-smoked, exercising 3 or more times per week, or a diagnosis of obesity, diabetes, asthma, or a mental health disorder. We then added interactions to the final model to estimate the effect modification between park access and race, education, and poverty status. These interactions were estimated in separate models to assess the effect modification of each variable without overfitting.

**Table 1:** Characteristics of Park Visitors (N = 3,605), by Blood Pressure (BP) Status, Southeastern Pennsylvania Household Health Survey, 2018

Characteristic	Normal BP, n (%)	High BP, n (%)	*P *value	Total, n (%)
	n = 2,131	n = 1,474		n = 3,605
**Have park**				
Yes	1,699 (79.7)	1,017 (69.0)	<.001	2,716 (75.3)
No	317 (14.9)	320 (21.7)		637 (17.7)
Yes, but not comfortable visiting	115 (5.4)	137 (9.3)		252 (7.0)
**Age, y**				
18–34	284 (13.3)	35 (2.4)	<.001	319 (8.9)
35–49	472 (22.2)	94 (6.4)		566 (15.7)
50–64	771 (36.2)	502 (34.1)		1,273 (35.3)
≥65	604 (28.3)	843 (57.2)		1,447 (40.1)
**Sex**				
Male	862 (40.5)	629 (42.7)	.18	1,491 (41.4)
Female	1,269 (59.5)	845 (57.3)		2,114 (58.6)
**Race**				
Black	323 (15.5)	399 (27.9)	<.001	722 (20.6)
White	1,604 (77.1)	942 (65.9)		2,546 (72.6)
Other	153 (7.4)	88 (6.2)		241 (6.9)
**Annual income less than 150% of federal poverty level**				
Yes	358 (16.8)	412 (27.9)	<.001	770 (21.4)
No	1,772 (83.2)	1,062 (72.1)		2,834 (78.6)
**Comorbidities (yes only)**				
Diabetes	170 (8.0)	442 (30.2)	<.001	612 (17.1)
Asthma	322 (15.1)	283 (19.2)	.001	605 (16.8)
Ever smoked	811 (38.3)	744 (50.8)	<.001	1,555 (43.4)
Mental health disorder	349 (16.5)	310 (21.1)	<.001	659 (18.4)
Obesity	497 (24.2)	586 (41.2)	<.001	1,083 (31.2)
**Social capital**				
Low	426 (22.3)	319 (25.3)	.09	745 (23.5)
Medium	1,009 (52.9)	663 (52.5)		1,672 (52.8)
High	472 (24.8)	280 (22.2)		752 (23.7)
Missing	224	212		436
**Education**				
Less than high school graduate	61 (2.9)	100 (6.8)	<.001	161 (4.5)
High school graduate, some college	902 (42.5)	793 (54.0)		1,695 (47.2)
College degree or more	1,160 (54.6)	576 (39.2)		1,736 (48.3)
**Marital status**				
Married or partnered	1,210 (57.1)	703 (47.8)	<.001	1,913 (53.3)
Single	470 (22.2)	298 (20.3)		768 (21.4)
Divorced or separated	196 (9.3)	179 (12.2)		375 (10.5)
Widowed	203 (9.6)	277 (18.8)		480 (13.4)
Other	40 (1.9)	14 (0.9)		54 (1.5)
Missing	12	3		15
**Servings of fruit and vegetables per day**				
0–2	1,081 (51.7)	865 (60.0)	<.001	1,946 (55.1)
** ≥**3	1,011 (48.3)	576 (40.0)		1,587 (44.9)
Missing	39	33		72
**Exercise ≥3 days per week**	1,309 (62.0)	711 (48.9)	<.001	2,020 (56.6)
**Missing**	18	20		38

We used multilevel models to account for the clustering inherent in the sampling design and to model the variation in HBP odds ratios (ORs) that exists between zip codes in the Philadelphia metropolitan area (19). SEPHHS used a complex sampling scheme, with post-stratification weights to allow for population-average effects reporting and for projection to population totals. Following Snijders and Bosker, we treated the sampling design as ignorable or conditional upon our modeling of the effect of zip code, and included covariates that were used for post-stratification (27). This contrasts with previous studies using SEPHHS data (22) where the results were presented as population-average effects using generalized estimating equations. Our rationale for using model-based rather than design-based inference was twofold: 1) our interest was in actually modeling the variation of effects among zip codes, and 2) the distribution of SEPHHS data does not include probability weights, which are necessary for design-based inference in the multilevel modeling context.

All analyses were performed in Stata 17.0 (Statacorp, LLC). A value of *P *<.05 was considered significant, and 95% CIs were reported.

## Results

Our population was predominantly older than 65 years (40.1%), female (58.6%), White (72.6%), married or partnered (53.3%), and had an annual income above 150% of the federal poverty level (78.6%). We saw significant differences between those who self-reported having and not having HBP. Among those who had access to a neighborhood park, 69.0% reported HBP, compared with 79.7% who reported not having HBP. A higher percentage of those with HBP were aged 65 years or older (57.2% with vs 28.3% without, *P* < .001), Black (27.9% with vs 15.5% without, *P* < .001), below 150% of the federal poverty level (27.9% with vs 16.8% without, *P* < .001), with diabetes (30.2% vs 8.0% without, *P* < .001), asthma (19.2% with vs 15.1% without, *P* = .001), a history of smoking (50.8% with vs 38.3% without, *P* < .001), with a diagnosed mental health disorder (21.1% with vs 16.5% without, *P* < .001), with obesity (41.2% with vs 24.2% without, *P* < .001), and with less than a high school education (6.8% with vs 2.9% without, *P* < .001) (Table 1). A lower percentage of those with HBP were married (47.8% married vs 57.1% without HBP, *P* < .001), ate 3 or more servings of fruits and vegetables per day (60.1% yes vs 51.7% without HBP, *P* < .001), and exercised 3 or more days per week (48.9% with vs 62.0% without HBP, *P* < .001).

Our random-intercept model confirmed significant variance among the zip codes in the odds of HBP (variance = 0.074, log likelihood test vs logistic [LR)] test *P* < .001). In the univariable model, those without a park had 68.0% higher odds of HBP (OR = 1.68; 95% CI, 1.4–2.0); those who had a park but were not comfortable visiting had 91% higher odds of HBP compared with those with a park (OR = 1.91; 95% CI, 1.5–2.5). After adjustment, the park categories remained significant: without a park, OR = 1.37; 95% CI, 1.1–1.7; with a park but not comfortable visiting, OR = 1.41; 95% CI, 1.03–2.0 (Table 2). Other significant variables in the final model included being aged 65 years or older (OR = 14.0; 95% CI, 9.1–21.4), of Black race versus White race (OR 2.0, 95% CI, 1.6–2.5), other race vs White (OR 1.45; 95% CI, 1.02–2.1), having diabetes (OR = 2.56; 95% CI, 2.1–3.2), asthma (OR = 1.26, 95% CI, 1.0–1.6), history of smoking (OR = 1.26; 95% CI, 1.1–1.5), having a mental health disorder (OR = 1.74, 95% CI, 1.4-2.2), or having obesity (OR = 2.0; 95% CI, 1.7–2.4). Higher education (college gradute or more) (OR = 0.63; 95% CI, 0.4–0.98) and exercising 3 or more times per week (OR = 0.79; 0.7–0.9) had lower odds of HBP. 

**Table 2:** Multilevel Models of the Association Between High Blood Pressure and Park Access Among Park Visitors (N = 3,605), Southeastern Pennsylvania Household Health Survey, 2018^a^

Fixed effects variable	Multivariable
**Park access**	
Yes, has park	1 [Reference]
No park	1.37 (1.1–1.7)
Yes, has park but not comfortable visiting	1.41 (1.03–2.0)
**Age, y**	
18–34	1 [Reference]
35–49	1.82 (1.1–2.9)
50–64	6.31 (4.1–9.6)
**≥**65	14.00 (9.1–21.4)
**Female (vs male)**	0.74 (0.6–0.9)
**Race**	
White	1 [Reference]
Black	2.00 (1.6–2.5)
Other	1.45 (1.02–2.1)
**Annual income less than 150% of federal poverty level**	1.26 (1.02–1.5)
**Comorbidities, yes**	
Diabetes	2.56 (2.1–3.2)
Asthma	1.26 (1.0–1.6)
Ever smoked	1.26 (1.1–1.5)
Mental health disorder	1.74 (1.4–2.2)
Obesity	2.00 (1.7–2.4)
**Education**	
Less than high school graduate	1 [Reference]
High school graduate, some college	0.81 (0.5–1.2)
College graduate or more	0.63 (0.4–0.98)
**Exercise ≥3 days per week**	0.79 (0.7–0.9)

Abbreviation: LR, log likelihood test versus logistic model.

a Values are odds ratio (95% CI). Random effects for zip code were zero for variance and LR.

Finally, the effect-modification analysis (Table 3) showed a strong effect for race and minimal effects for education or poverty levels. Compared with White participants with access to a park, White participants without a park had higher odds of HBP (OR = 1.39; 95% CI, 1.1–1.8), but those not comfortable visiting their park were not significantly different. We observed an increasing OR for Black participants with a park (OR = 1.95; 95% CI, 1.5–2.5), without a park (OR = 2.69; 95% CI, 1.8–4.1), and with a park but not comfortable visiting it (OR = 3.5; 95% CI, 2.0–6.2) compared with White participants with a park. Respondents of other races with a park had higher odds of HBP compared with White participants with a park (OR = 1.58; 95% CI, 1.04–2.4). The effect-modification analyses for education, poverty status, and park access revealed similar results to the main effects, and no clear pattern of effect. For instance, odds of HBP were higher for almost all categories where the group did not have a park or were not comfortable visiting the park, but the ORs were similar in magnitude. The one exception was among college graduates who had a park but were not comfortable visiting it had an OR of 2.2 (95% CI, 1.2–4.1) compared with college graduates with a park (Table 3).

**Table 3:** Effect Modification of Association Between High Blood Pressure and Park Access Among Park Visitors (N = 3,605), by Race, Education, and Poverty Status^a^, Southeastern Pennsylvania Household Health Survey, 2018

Variable	Has park	No park	Has park but not comfortable visiting
**Race**			
Black	1.95 (1.5–2.5)	2.69 (1.8–4.1)	3.5 (2.0–6.2)
White	1 [Reference]	1.39 (1.1–1.8)	1.3 (0.9–2.0)
Other	1.58 (1.04–2.4)	1.7 (0.8–3.6)	1.7 (0.7–4.1)
**Education**			
College graduate	1 [Reference]	1.5 (1.1–2.1)	2.2 (1.2–4.1)
High school graduate	1.4 (1.1–1.7)	1.8 (1.3–2.4)	1.7 (1.1–2.5)
Less than high school graduate	1.9 (1.1–3.4)	2.0 (0.9–4.1)	1.7 (0.5–5.4)
**Annual income less than 150% of federal poverty level**			
No	1 [Reference]	1.4 (1.1–1.8)	1.6 (1.1–2.4)
Yes	1.3 (1.1–1.7)	1.6 (1.1–2.4)	1.6 (0.9–2.6)

a Adjusted for age, sex, race, poverty status, diabetes, asthma, ever smoked, mental health, education, exercise, zip code. Values are odds ratio (95% CI).

## Discussion

Proximity to green spaces, including parks, has been shown to have protective effects on HBP and other chronic health conditions. Our study supports these findings and additionally shows that feeling comfortable visiting a neighborhood park is also associated with HBP. Our findings that not having a park or not feeling comfortable visiting a park were both associated with higher odds of HBP imply that if residents do not feel comfortable visiting a neighborhood park, they may not access the health benefits associated with these green spaces. Our study adds to the results of existing studies that reported less park activity because of high crime or concerns about safety (9,12,13), but our study links these findings specifically to a higher risk of HBP.

We used multilevel models to account for the clustering present in the data. These also allowed for modeling the variance in the odds of HBP across zip codes in the Philadelphia area, in which we found significant variation. Unlike the Koh study (22), where population-average effects were presented, our reported effects represent the effect in a typical zip code (ie, one close to the mean on all measures). Though we found no evidence for modeling differences in the effects across zip code, our approach is informative for 2 reasons. First, failure to adjust for clustering in a sample leads to biased standard errors, which will in turn affect significance tests and CIs. Several methods allow for analyzing clustered data, and we chose multilevel modeling because we were interested in the distribution of effects across zip codes, rather than constructing a population average. Second, the advantage of our approach is it allows for interactions of variables across the levels of the design. For example, future work could examine whether zip codes with more or less green space have higher or lower ORs of HBP across racial categories. Though it is possible to test for such effects in population-average models, modeling the multilevel structure of the data yields a more nuanced understanding of such cross-level interactions (20). To fully account for the variation in HBP by zip code, future studies should include additional data aggregation with available zip-code–level variables (eg, available green space, access to healthy foods).

Despite the lack of zip-code–level predictors, we did perform several individual-level subgroup analyses to determine whether the effects of park access and perceptions of safety varied by race and SES. We found that for Black participants, the odds of HBP increased with a lack of park access and a perception of being uncomfortable visiting a neighborhood park. The odds of HBP for Black participants with a park were twice as high as for White participants with a park. Additionally, the odds of HBP for Black participants were 2.7 times higher with no park and 3.5 with a park but not feeling comfortable visiting it. Although these ratios indicate a racial disparity, the disparity is lessened by the presence of a park that participants are comfortable visiting. The effect-modification analysis for education and poverty status showed no clear pattern of modification. Most groups of similar education and poverty levels who did not have park access or were not comfortable visiting a park, had ORs of similar magnitude and were similar to the main effects. The one exception was a 2.2 times higher odds for college graduates with a park who were not comfortable visiting their park.

Our findings have some similarities with other studies that have used SEPHHS. Koh et al (22) found that odds of HBP decreased as education and age increased and were higher for Black than for White participants. They used objective measurements of tree-canopy cover and proximity to green spaces and found no effect modification in HBP disparity by levels of tree-canopy cover. This could have resulted from their choice of analysis, which did not model the multilevel nature of the data. Our study also showed a similar disparity in the odds of HBP across levels of education and park access. Knobel et al (21) found that perception of park access was protective of HBP for non-Hispanic Black participants. Our findings confirm this but add the effect modifications of park access and perceptions of safety.

Because historic redlining is associated with increased CVD (28), increased odds of HBP among Black participants with lower perceived access to parks elucidates a potential link to this practice. Redlining is responsible for racially segregated neighborhoods and de-investment in Black communities, and most Black residents in the Philadelphia metropolitan region live in racially segregated neighborhoods (18). These communities are also subject to the development of large industrial or business complexes and highways that decrease green spaces and are linked to higher CVD risk (29). A recent study demonstrated that Black residents living in historically redlined neighborhoods had a higher prevalence of CVD risk factors, including HBP, than those living in nonredlined neighborhoods (30). Future studies are needed to research the relationship between redlined neighborhoods, green space access, and chronic disease. Multilevel modeling allows for better examination of these structural effects than population-average models.

In addition, the highest odds of HBP were observed among Black participants who had a park but were not comfortable visiting it. Although the survey did not ask why they were not comfortable, it could be because of feeling unsafe. A recent Gallup survey reported that Black Americans feel less safe in their communities compared with other racial groups, and, notably, 51% of Black women felt unsafe walking alone in their neighborhood (31). Feelings of being unsafe may contribute to allostatic load, a condition characterized by McEwen and Seeman (32) as the lifetime accumulation of stress that affects health. Allostatic load may come from stress due to trauma or abuse; environments like home, school, or neighborhoods; and for Black Americans, from experiences with discrimination (33). The lifetime stress experienced by Black Americans has been linked to the health disparities seen between Black and White Americans at younger ages and is independent of SES (33). Black participants who feel uncomfortable visiting a park in their neighborhood may be experiencing many other stressors that contribute to higher odds of HBP. Conversely, for Black participants who have a park in their neighborhood and feel comfortable visiting it, the odds of HBP are higher than for White participants with a park, but 1.5 times lower than those who have a park but do not feel comfortable visiting. This finding suggests that parks in a neighborhood and perceptions of safety may lessen health disparities for Black residents, which is evidence of an equigenic effect.

These findings could inform public health departments and organizations about additional avenues for reducing health disparities and HBP in general. Past studies have suggested that violent crime, lighting, broken glass, and busy roads are all concerns related to perceived safety and park use (9–13). Public health officials and city planners, working together, could create plans to alleviate these safety concerns. Providing park access to neighborhoods may also be an important mechanism for reducing HBP.

Our study had several limitations. First, it was a cross-sectional study that lacked temporality. We cannot say with certainty that perceptions of park access led to a reduction or increase in HBP. It is unknown whether a participant had HBP at the time of the study or whether they only had a history of HBP. We also do not know if those who answered “no” to having access to a park really did not have a park in their neighborhood. In addition, we can only make inferences about why participants did not feel comfortable visiting a park or outdoor space in their neighborhood. We can only generalize the results to people living in the Philadelphia metropolitan region, and the results do not represent changes that may have arisen because of the pandemic. In addition, some of the ORs were close to 1, which might be due to low numbers in some cells. The strengths of this study include the multilevel modeling that handled the clustered nature of observations by allowing variation in HBP by zip code and the number of confounders for which we controlled. We were also able to infer that not having a park or having a park but not feeling comfortable visiting it were both associated with higher odds of HBP; therefore, the physical presence of a park is not enough to encourage park visits. Thus, community planners should keep in mind perceptions of safety when planning new or evaluating existing green spaces.

Perceptions of park access are important for the health of people in a community. Our study showed an association between HBP and not having a park or having a park but not feeling comfortable visiting it. We observed an effect modification by race in which Black participants with a park, without a park, or with a park but not feeling comfortable visiting it had increased odds of HBP. Parks in neighborhoods can positively affect HBP, but addressing safety concerns and ensuring equitable access is essential to maximize their health-promoting potential.
